# Frameshift Variant in MFSD12 Explains the Mushroom Coat Color Dilution in Shetland Ponies

**DOI:** 10.3390/genes10100826

**Published:** 2019-10-19

**Authors:** Jocelyn Tanaka, Tosso Leeb, James Rushton, Thomas R. Famula, Maura Mack, Vidhya Jagannathan, Christine Flury, Iris Bachmann, John Eberth, Sue M. McDonnell, Maria Cecilia T. Penedo, Rebecca R. Bellone

**Affiliations:** 1 Veterinary Genetics Laboratory, School of Veterinary Medicine, University of California, Davis, CA 95616, USA; jltanaka@ucdavis.edu (J.T.); Maura.Mack@colostate.edu (M.M.); mctorrespenedo@ucdavis.edu (M.C.T.P.); 2 Institute of Genetics, University of Bern, 3001 Bern, Switzerland; tosso.leeb@vetsuisse.unibe.ch (T.L.); vidhya.jagannathan@vetsuisse.unibe.ch (V.J.); 3 Rowe Equine Ltd, Wotton Under Edge, GLOS GL12 7PP, UK; jamesrushton@rowevetgroup.com; 4 Department of Animal Science, University of California, One Shields Ave, Davis, CA 95616, USA; trfamula@ucdavis.edu; 5 School of Agricultural Forest and Food Sciences, Bern University of Applied Sciences, 3052 Zollikofen, Switzerland; christine.flury@bfh.ch; 6 Agroscope, Swiss National Stud Farm, 1580 Avenches, Switzerland; iris.bachmann@agroscope.admin.ch; 7 College of Agriculture, Food and Environment, University of Kentucky, Lexington, KY 40546, USA; john.eberth@uky.edu; 8 Havemeyer Equine Behavior Program, University of Pennsylvania School of Veterinary Medicine, New Bolton Center Department of Clinical Studies, Kennett Square, PA 19348, USA; suemcd@vet.upenn.edu; 9 Population Health and Reproduction, School of Veterinary Medicine, University of California, Davis, CA 95616, USA

**Keywords:** *Equus caballus*, coat color, pigmentation, *MFSD12*, dilution

## Abstract

Mushroom is a unique coat color phenotype in Shetland Ponies characterized by the dilution of the chestnut coat color to a sepia tone and is hypothesized to be a recessive trait. A genome wide association study (GWAS), utilizing the Affymetrix 670K array (MNEc670k) and a single locus mixed linear model analysis (EMMAX), identified a locus on ECA7 for further investigation (*P*_corrected_ = 2.08 × 10^−10^). This locus contained a 3 Mb run of homozygosity in the 12 mushroom ponies tested. Analysis of high throughput Illumina sequencing data from one mushroom Shetland pony compared to 87 genomes from horses of various breeds, uncovered a frameshift variant, p.Asp201fs, in the *MFSD12* gene encoding the major facilitator superfamily domain containing 12 protein. This variant was perfectly concordant with phenotype in 96 Shetland Ponies (*P* = 1.15 × 10^−22^), was identified in the closely related Miniature Horse for which the mushroom phenotype is suspected to occur (f_mu_ = 0.02), and was absent in 252 individuals from seven additional breeds not reported to have the mushroom phenotype. *MFSD12* is highly expressed in melanocytes and variants in this gene in humans, mice, and dogs impact pigmentation. Given the role of *MFSD12* in melanogenesis, we propose that p.Asp201fs is causal for the dilution observed in mushroom ponies.

## 1. Introduction

**Figure 1:**
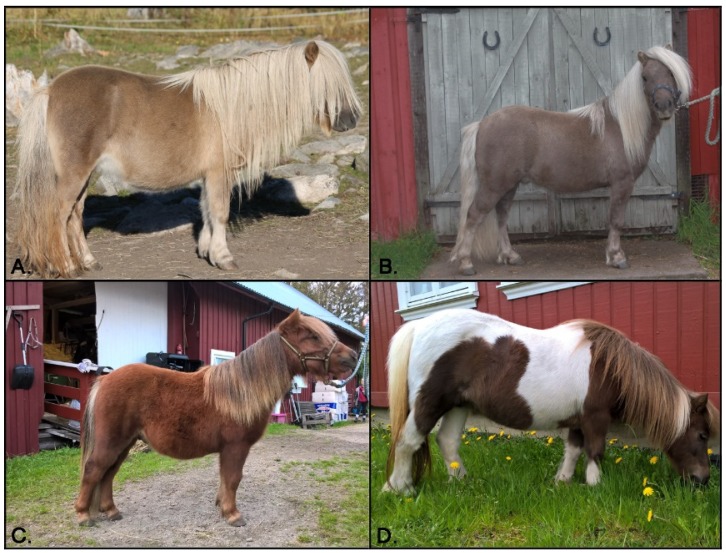
Mushroom and chestnut coat color phenotypes. The mushroom dilution on a chestnut background results in a dilute sepia shade ranging from light (**A**) to dark (**B**) and is often accompanied by a flaxen (lighter) mane and tail. Chestnut Shetland pony without the mushroom dilution displaying a typical red chestnut base coat color (**C**). Chestnut Shetland Pony also showing the tobiano white spotting pattern (**D**). Photo credits (**A**,**B**) Maria and Joana Tammi (**C**,**D**) Christine Mirjam Sorli.

Pigmentation is influenced by numerous genes controlling both the type and quantity of melanin produced in the skin and hair of animals. To date, over 300 genes have been described to be involved in mammalian pigmentation [1]. Genetic variation in these genes contributes to both base color, shade, and intensity of pigmentation. Two types of melanin are produced in mammals; eumelanin, or brown/black pigment, and pheomelanin, or red/yellow pigment. The overall phenotype depends on the quantity and distribution of these two pigments. In horses, three main base coat colors, bay, black, and chestnut exist and these are largely controlled by the two genes *ASIP* and *MC1R* encoding agouti signaling protein and melanocortin 1 receptor, respectively. The agouti locus (*ASIP*) controls the location and pattern of eumelanin, with the dominant (*A*) allele causing the restriction of eumelanin to a horses’ points (mane, tail, lower legs, ears, and muzzle) seen on bay horses. The recessive (*a*) allele, caused by an 11 bp deletion (c.191_201del) at the agouti locus does not restrict eumelanin, resulting in a uniformly black horse [2]. The extension locus, encoding MC1R, is epistatic; horses homozygous for the loss of function allele (*e*) in *MC1R* (c.248C>T, p.S83F) only produce pheomelanin, resulting in a chestnut coat color (red coat, mane, and tail), regardless of the genotype at the agouti locus (Figure 1C,D). 

Throughout the development of horse breeds, breeders have selected for variations in the amount and type of pigment, as well as white patterning. Six alleles (cream, pearl, sunshine, champagne, dun, silver) have been described in the horse that dilute either pheomelanin, eumelanin or both. Three of these alleles are caused by variants in *SLC45A2*; cream (*Cr*) c.457G>A, pearl (*Prl*) c.985G>A, sunshine (*Sun*) c.586G>A are thought to disrupt protein function and subsequent trafficking of molecules to the melanosomes resulting in a reduction of pigment production [3,4,5]. Both *Prl* and *Sun* follow a recessive mode of inheritance and dilute both eumelanin and pheomelanin, while *Cr* shows an incomplete dominant mode of inheritance and affects pheomelanin in a heterozygous state and both pheomelanin and eumelanin in a homozygous state [3]. The champagne (*Ch*) coat color dilution caused by (*SLC36A1* c.188C>G) is a completely dominant allele that dilutes both pheomelanin and eumelanin. The *SLC36A1* c.188C>G missense variant is hypothesized to affect the development of early melanosomes by altering the pH and thus the pathway regulating melanosomal development [6]. Dun is another known coat color dilution that affects both pheomelanin and eumelanin and is characterized by a dilute coat accompanied by primitive markings (dark dorsal stripe, leg stripes, shoulder bars, and dark markings on the forehead known as cobwebbing). Dun is believed to be the ancestral phenotype and a 1617 bp deletion downstream of T-box 3 transcription factor (*TBX3*) explains the non-dun phenotype. The non-dun allele results in differential expression of *TBX3* that alters pigment deposition along the longitudinal axis of the hair follicle [7]. The silver dilution (*Z*) is inherited in a dominant fashion and affects eumelanin only, resulting in a dilute coat with a flaxen mane and tail in both bay and black horses, while the coats of chestnut horses appear phenotypically normal. The *Z* allele caused by the *PMEL17* p.Arg625Cys variant is proposed to affect the normal deposition of eumelanin in maturing melanosomes, creating a dilute phenotype [8,9]. The *Z* allele is also associated with the pleiotropic disorder, multiple congenital ocular anomalies (MCOA). MCOA is an inherited set of ocular anomalies where heterozygotes typically have temporal iris, ciliary body, or retinal cysts and homozygotes have the more severe phenotype that includes cysts and other bilateral anomalies including cornea globosa, uveal cysts, iris stromal hypoplasia, persistent miosis, and cataracts [9].

Mushroom is another dilution phenotype for which the causal variant has not yet been identified. This phenotype has been reported in the Shetland Pony on the chestnut background, and is characterized by a dilute, sepia coat. The dilute body was also noted to be accompanied by a lighter mane and tail, often referred to as flaxen (Figure 1A,B). The body shade can range from a lighter yellow, appearing similar in phenotype to that of one copy of the cream dilution on a chestnut background (palomino), to a deeper taupe shade, phenotypically similar to horses carrying the silver allele. It is unknown if the mushroom dilution causes ocular anomalies analogous to that of silver. Investigating breeding records, supports an autosomal recessive mode of inheritance for the mushroom phenotype [10]. A previous study determined that 50 out of 51 Shetland ponies suspected to have the mushroom phenotype were homozygous (*e/e*) at *MC1R*, but wildtype at *PMEL17* (silver). The remaining pony was determined to be a silver bay (*E/e, A/A, Z/z*) [11]. These data support a genetic mechanism for mushroom distinct from that of silver with the mushroom dilution reducing pheomelanin rather than eumelanin. In this study, we performed a genome-wide association study (GWAS) followed by whole genome sequencing (WGS) to identify a variant causing the mushroom phenotype.

## 2. Materials and Methods 

### 2.1. Horses and Phenotyping

Hair follicles or whole blood were collected from a total of the 113 Shetland Ponies whose proposed coat color phenotype was either mushroom or chestnut (non-mushroom). Bilateral photographs clearly displaying coat color were provided by owners and were used to phenotype horses for mushroom. Mushroom individuals displayed a clear sepia toned coat as well as a lighter mane and tail. Non-mushroom horses were identified as those horses with a chestnut phenotype (red body and red mane and tail) with no evidence of dilution in the coat or in the mane and tail. Since varying shades of a mushroom coat color can resemble known coat color dilutions, particularly the cream and silver dilutions, individuals were screened for known dilution alleles (cream, pearl champagne, dun and silver) and individuals with any dilution allele were excluded (*N* = 17 individuals) from the GWA and validation studies leaving 96 individuals. Additionally, since the mushroom phenotype was previously reported to only occur on a chestnut background, horses were also genotyped for *ASIP* and *MC1R* and only ponies with the (*e/e*) genotype were included in the initial phase of the study to identify the locus for mushroom. Genotyping for known coat color dilutions and for *MC1R* and *ASIP* was performed by the Veterinary Genetics Laboratory, UC Davis diagnostic testing services.

DNA samples from eight additional horse breeds, closely or distantly related to the Shetland Pony based on a phylogenetic analysis [12], were also included in this study to investigate potential causal variants. These breeds included Icelandic Horses (*N* = 29), Miniature Horses (*N* = 129), Belgian (*N* = 33), Thoroughbred (*N* = 31), Quarter Horses (*N* = 32), Arabians (*N* = 35), Rocky Mountain Horses (*N* = 59), and Friesians (*N* = 32). DNA from an additional unrelated sample set of Shetland Ponies (*N* = 177) was used to estimate the frequency of the mushroom allele in the breed, as well as to investigate the potential impact of the allele on other base colors. DNA from these samples was isolated from hair collected for this study or were samples previously banked in the Bellone Equine Research Laboratory.

Genomic DNA utilized for the GWAS was isolated from hair roots using the Gentra Puregene DNA isolation Kit as previously described [13]. DNA used for genotyping and validating variants of interest was isolated according to a crude hair lysis protocol [14]. Additionally, genomic DNA for WGS was isolated from whole blood using the Gentra Puregene DNA isolation Kit as previously described in [15].

### 2.2. Genome Wide Association Study

A GWAS with 24 horses was conducted with mushroom (*N* = 12) and chestnut (*N* = 12), non-mushroom ponies. The Axiom Equine Genotyping 670K array [16] was utilized and samples were processed and genotyped by Geneseek (Lincoln, NE, USA). GWAS data were remapped to Equcab3 [17,18]. Analysis and visualization of data were performed using Golden Helix SNP & Variation Suite v8 (Golden Helix, Bozeman, MT, USA). Standard quality control filters were applied (sample call rate > 95%, SNP call rate > 90%, minor allele frequency > 5%) leaving 356K SNPs for analysis. To maximize the power to detect an association for a recessive trait in the relatively small sample set, a χ2 basic allelic association test was performed. To investigate cryptic relatedness in our sample set as evidenced by the genomic inflation factor (λ = 1.11), genome wide identity by descent (IBD) was calculated and visualized on a heat map. A single locus mixed linear model (SLMM) approach was employed to correct the detected relatedness in our sample set. The SLMM utilizes an identity by state (IBS) to estimate population structure and Efficient Mixed-Model Association eXpedited (EMMAX) is utilized to calculate P-values using an F-test. Loci reaching a strict Bonferroni correction to correct for multiple testing were further considered (*P* < 1.4 × 10^−7^). Since the trait was hypothesized to be recessive, haplotypes were evaluated manually to investigate runs of homozygosity.

### 2.3. Validation of Association

To confirm the association on ECA7 reaching genome wide significance, eight markers from this locus spanning 326 kb and two flanking SNPs were genotyped in 45 additional ponies (28 mushroom, 17 chestnut). These variants were genotyped using an Agena Bioscience MassArray assay and performed at the Veterinary Genetics Laboratory, UC Davis. Markers tested and primer sequences can be found in Table S1. This haplotype was also screened in four additional breeds, Miniature Horse (*N* = 30), Icelandic Horse (*N* = 31), Thoroughbred (*N* = 24), Quarter Horse (*N* = 22), as well as an additional sample set of Shetland Ponies (*N* = 33).

### 2.4. Whole Genome Sequencing and Variant Investigation

Whole genome sequencing of one mushroom Shetland Pony was performed at the University of Bern, as part of an effort to better characterize diversity in the equine genome [19]. The genomes of 88 horses from 25 various breeds were evaluated. An illumina TruSeq PCR free DNA library with 350 bp insert size was prepared from a mushroom Shetland Pony sample. This library was sequenced on an Illumina HiSeq 3000 instrument with 2 × 150 bp paired-end reads to an average depth of 19X coverage. Sequencing data were filtered for quality, with low quality reads discarded, and aligned to the horse reference genome via the pipeline Speedseq [20]. This pipeline utilizes Burrows-Wheeler Aligner [21] to align the fastq files to the reference genome, and subsequently pipes the data to SAMBLASTER [22] and SAMBAMBA [23] to mark and store duplicate reads [19]. Variants were called using the Genome Analysis Toolkit HaplotypeCaller (GATK) [24], and annotated via Snpeff [25]. Variant prioritization was focused on the associated 3 Mb run of homozygosity on ECA7 and variants homozygous in the Shetland but absent in all 87 other horses were considered for further evaluation.

### 2.5. Validation of Causative Variant

To confirm the MFSD12 c.600dupC variant identified by the whole genome sequencing analysis, Sanger Sequencing was performed with primers designed via Primer3 [26,27] and NCBI Primer Blast [28] (Table S2). The PCR protocol was performed with a total volume of 20 ul using 5.0 pmol of primers, 25 ng of DNA, 1 × PCR buffer with 2.0 mM MgCl_2_, 1 mM dNTP, and 0.1 µl FastStart Taq DNA polymerase (Roche Applied Science, Indianapolis, IN, USA).

PCR products were visualized on a 1% EtBr agarose gel to verify correct product size before sequencing. The amplicons were purified using an EdgeBio Quickstep 2 PCR purification kit following the manufactures protocol (EdgeBio, Gaithersburg, MD, USA). Amplicons were subsequently sequenced using BigDye Terminator v1.1 and products resolved on an ABI 3730 Genetic Analyzer (Applied Biosystems, Inc. at ThermoFisher Scientific, Waltham, MA, USA). Sequencing data were aligned and analyzed for variants using Sequencher v5.4 (Gene Codes, Ann Arbor, MI, USA).

Ten mushroom Shetland Ponies, nine chestnut Shetland Ponies, two Icelandic Horses and one Miniature Horse were sequenced. A multiple sequence alignment of all 22 horses was performed using Sequencher (Gene Codes Ann Arbor, USA). Additional Shetland Ponies were genotyped by PCR assay for a total of 96 individuals (*N* = 45 mushroom, *N* = 51 chestnut). Horses from 8 additional breeds, Icelandic Horses (*N* = 29), Miniature Horses (*N* = 129), Belgian (*N* = 33), Thoroughbred (*N* = 31), Quarter Horses (*N* = 32), Arabians (*N* = 35), Rocky Mountain Horses (*N* = 59) and Friesians (*N* = 32), as well as an additional sample set of Shetland Ponies of various coat colors (*N* = 177) were also genotyped for this marker and similarly visualized on an ABI 3730 Genetic Analyzer. A χ^2^ test was used to calculate association between MFSD12 c.600dupC and the mushroom phenotype. Additionally, the allele frequency of MFSD12 c.600dupC was calculated for each population screened for the variant.

### 2.6. Ophthalmic Examination

To investigate if the mushroom dilution was associated with any ocular anomalies, a board-certified veterinary ophthalmologist examined the eyes of 20 Shetland Ponies (*N* = 9 mushroom, *N* = 11 non-mushroom). These ponies were first coat color phenotyped by bilateral photograph and subsequently DNA tested for all known coat color variants, at the Veterinary Genetics Laboratory, UC Davis, so that other coat color variables could be considered in the statistical analysis as described below. Specifically, genotypes at both the tobiano locus (To), which is caused by a large inversion 70 kb downstream of tyrosine-protein kinase (KIT) [29], and the splash white 1 locus (SW1), an insertion in an microphthalmia-associated transcription factor (MITF) promoter [30] were considered as white patterning was observed in the sample of Shetland Ponies. To confirm the mushroom phenotype, these samples were also genotyped for the mushroom associated variant MFSD12 c.600dupC. A complete ophthalmic examination was performed, evaluating the eyelid, conjunctiva, cornea, anterior chamber, iris, corpora nigra, lens, vitreous, retina, interocular pressure (IOP), and pigmentation of the anterior and posterior uvea. This study was approved by the UC Davis IACUC committee under the protocol number (#19205). 

The pigmentation of the posterior uvea was categorized as being either pigmented or hypopigmented. The pigmentation of the posterior uvea was classified as hypopigmented if the choroidal vessels were visible, and normally pigmented if the vessels were not visible against the non-tapetal fundus. The anterior uveal pigment was categorized as hyperpigmented, pigmented or hypopigmented as compared to the characteristic typical light brown iris color. Ponies with a light brown iris color were categorized as having normal pigment, those ponies with a more intense shade of brown were categorized as hyperpigmented. Ponies categorized as having hypopigmentation displayed a lighter shade of brown. The pigmentation of the posterior and anterior uvea was evaluated to determine if there was a significant statistical difference between mushroom and non-mushroom individuals. Both a Fisher’s exact test as well as a Bayesian analysis utilizing a logistic regression in the R package rstanarm, were applied to the data. Specifically, in the Bayesian analysis, a “leave one out” (looic) strategy was used to explain the anterior and posterior pigmentation in relation to the individuals’ coat colors (mushroom, cream, tobiano, splash white 1), as coat color dilutions as well as white spotting patterns can have an impact on ocular pigmentation [6,30]. 

### 2.7. Data Availability

MAP and PED files from this study are available at Open Science Framework (OSF) https://osf.io/hdr9y/. Whole genome sequencing data was submitted to the European Variant Database (EVA) under the project accession PRJEB28306 [23].

## 3. Results

### 3.1. GWAS

**Figure 2:**
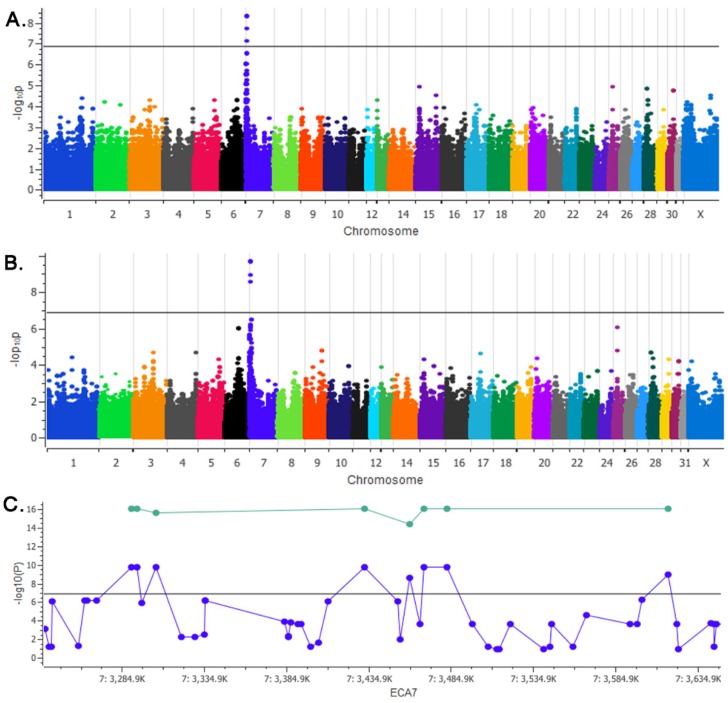
A genome wide association study (GWAS) identifies the locus for mushroom phenotype in Shetland Ponies. (**A**) Manhattan Plot for the χ^2^ basic allelic association test. (**B**) Single locus mixed linear model (SLMM) utilizing an F-test to calculate p-values. (**A**,**B**) Plotted on the y-axis are the -log 10P values calculated for each test against the chromosomes plotted on the x-axis. The horizontal black line across each plot represents Bonferroni significance (*P* < 1.4 × 10^−7^). (**C**) A 326 kb region on ECA7 reaching genome wide significance. Plotted in blue are the data points from the SLMM GWAS analysis (*N* = 24). Plotted in green are the eight SNPs from this region genotyped in our replication sample set, displaying the combined p-values under a recessive model for the GWAS and replication sample sets (*N* = 40 mushroom ponies, *N* = 29 chestnut).

A χ2 basic allelic association identified a single locus on ECA7 spanning 3 Mb that reached genome wide significance (*P*_corrected_ = 4.75 × 10^−9^) (Figure 2A). While reaching Bonferroni significance, genomic inflation was high (λ = 1.11). Investigating cryptic relatedness in our GWAS sample set by pairwise identity by descent (IBD) values identified one pair of chestnut and one pair of mushroom ponies sharing up to 52% of their genome, likely contributing to the observed genomic inflation (Figure S1). 

After correcting for relatedness, using a single locus mixed linear model employing an F-test (EMMAX analysis), the locus on ECA7 was further supported (Figure 2B, *P* = 2.08 × 10^−10^). The eight most concordant SNPs within this locus spanned 326 kb. Further examination of the haplotype of all mushroom samples in the GWAS data set identified a 3 Mb run of homozygosity in all 12 mushroom samples (ECA7:729453–3671999). Validating the eight most concordant GWAS SNPs and two additional SNPs flanking the ROH in 45 additional ponies (28 mushroom and 17 chestnut) confirmed the association to this region (Figure 2C, *P* = 1.97 × 10^−11^) (Table S1). Specifically, the 326 kb haplotype was perfectly concordant in all samples tested (*N* = 69, *P*_combined_ = 9.85 × 10^−17^, Table 1). This mushroom associated haplotype was subsequently screened in four additional breeds Miniature Horse (*N* = 30), Icelandic Horse (*N* = 31), Thoroughbred (*N* = 24), Quarter Horse (*N* = 22) as well as in an additional sample set of Shetland ponies (*N* = 33). Two Icelandic Horses and one Miniature Horse were found to be homozygous for the mushroom associated haplotype. The haplotype was identified in a heterozygous state in six Quarter Horses and eight Thoroughbreds (Table 2).

To identify candidate causal variants, the 3 Mb run of homozygosity associated with the mushroom phenotype, was investigated by comparing whole genome sequence data from this region in one mushroom Shetland Pony to that of 87 genomes from 24 other breeds [19]. Only one variant in the major facilitator superfamily domain containing 12 gene (MFSD12) NC_009150.3: g.2544512dupC (XM_023646425.1:c.600dupC, p.Asp201fs) was identified that was homozygous in the mushroom sample and absent from all other breeds. The variant was predicted by SnpEff to have a high functional consequence. If translated, MFSD12 c.600dupC would cause a frameshift and subsequent truncation of the protein by 277 amino acids.

MFSD12 c.600dupC was validated via Sanger sequencing 19 additional Shetland Ponies (*N* = 10 mushroom and *N* = 9 chestnut). All 10 mushroom samples were homozygous for the mutant allele (Mu), three chestnuts were heterozygous and four chestnuts (non-mushroom) were homozygous for the reference allele. Genotyping 96 Shetland Ponies (45 mushroom and 51 chestnut), by a PCR assay, identified the genotypes for this variant were perfectly concordant with the mushroom phenotype (*P* = 1.15 × 10^−22^) (Table 3). 

Five hundred and fifty-seven horses from eight additional breeds, as well as a random sample set of Shetland Ponies with diverse coat colors were genotyped for this variant. The allele frequency in the Shetland Pony (*N* = 177) was estimated to be 0.12 (Table 4). The allele was only detected in the Shetland and the Miniature Horse breeds (Table 4), providing further support that this is the causal variant. Shetland Ponies served as a foundation breed for the Miniature Horse [31] and the two breeds are often crossbred so it is not surprising that the Miniature Horse had a low allele frequency of 0.02 for the mushroom variant, although no Miniature Horses were homozygous for the mushroom allele, suggesting the mushroom phenotype is rare in this breed (Table 4). 

**Figure 3:**
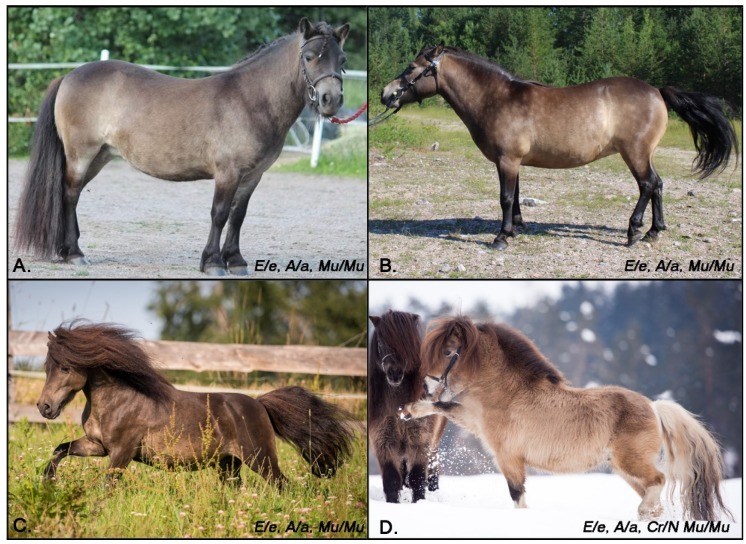
Bay mushroom Shetland Ponies. (**A**–**C**) Three bay ponies homozygous for the mushroom variant showing a coat lacking the red hue as typically seen in bay horses without the mushroom dilution. These bay mushroom ponies have normally pigmented mane and tail with countershading on the shoulders neck and head of these ponies. (**D**) Buckskin homozygous for the mushroom variant. The countershading is consistent with other “bay mushroom” ponies though the combination of mushroom and cream is likely contributing to the lighter shade in the coat of this pony as compared to the other bay mushrooms. Photo credits (**A**) Maria and Joana Tammi (**B**) Mira Ruotsalainen (**C**) and (**D**) Katrin Lach and Claudia Rahlmeier

Interestingly, in testing the Shetland sample set with diverse coat colors, seven ponies genotyping as bay or black based on the MCIR and ASIP genotypes were also homozygous for the mushroom allele (Mu/Mu) (Table 5). Inspection of photographs of these ponies identified altered phenotypes. Specifically, the six Shetland Ponies that genotyped as bay (E/_ A/_) and were also (Mu/Mu) were observed to have a coat that was less red than expected for bay horses (Figure 3). These six ponies showed consistent countershading with dark shoulders, necks, and heads. These findings support the hypothesis that the mushroom dilution reduces pheomelanin production, leaving the manes and tails of these bay ponies black and undiluted while the body hairs appeared to lack the red hue of normal bay horses and were more sepia in tone. Given the altered phenotype, we therefore propose to call the mushroom dilution on a bay background, bay mushroom. Additionally, one of these bay mushroom ponies was also heterozygous at the cream locus (buckskin), and was phenotypically more dilute than the bay mushroom ponies without any copies of the cream dilution. This suggests that on a bay base coat, the cream and mushroom dilutions cause a more dilute phenotype. The one black pony identified as being homozygous for the mushroom variant did not appear to have any dilution in coat, mane or tail as would be expected if mushroom only dilutes pheomelanin. 

**Figure 4:**
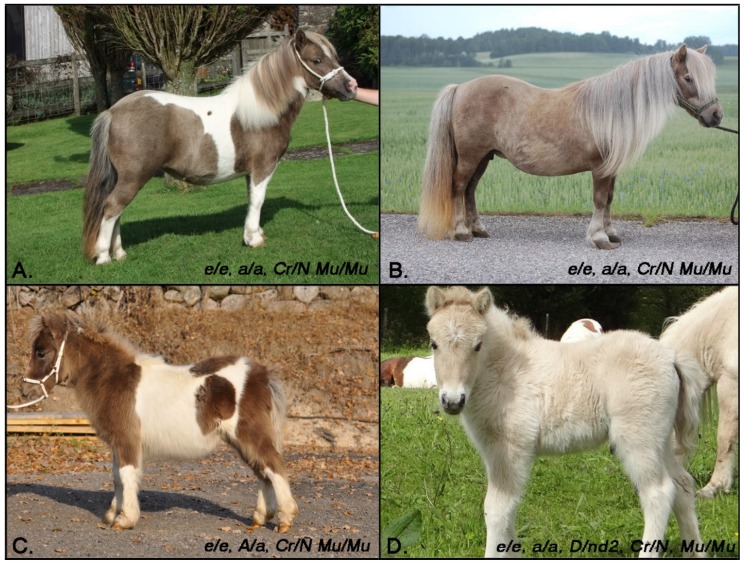
Palomino Mushroom Shetland Ponies. (**A**–**C**) Three palomino mushroom ponies (chestnut background and heterozygous at the cream locus), displaying a phenotype consistent with other mushroom individuals without the cream dilution ranging in shade of mushroom phenotype. (**D**) Dunalino (palomino with the dun dilution) homozygous for the mushroom dilution, displaying an extremely dilute phenotype likely caused by a combination of dun and cream. On all four ponies the head appears to be a darker shade, similar to what is seen on the bay mushroom ponies. Photo credits (**A**) David Hodge. (**D**) Marc and Cecile Letouzé.

Five ponies were identified as Mu/Mu, as well as heterozygous at the cream locus (N/Cr) (Table 5). These five ponies displayed a coat color that was not distinguishable from the mushroom phenotype (Figure 4) suggesting that the cream allele does not further dilute the mushroom phenotype on a chestnut background, different to the additive effect of the cream and mushroom dilutions observed on a bay background (Figure 3D). No individuals in this study were Mu/Mu, Cr/Cr, so it is unknown if these animals display a more dilute phenotype. Additionally, one Mu/Mu, N/Cr pony was identified as heterozygous for the dun dilution (D/nd2). This pony displayed an extremely dilute phenotype, though we hypothesize this phenotype was due to the presence of the dun dilution in combination with the cream allele (Figure 4D). All five chestnut-based ponies (e/e at the extension locus) homozygous for the mushroom dilution and heterozygous for the cream dilution displayed countershading with a darker head similar to what was observed in the bay Mu/Mu ponies (Figure 4).

### 3.2. Ophthalmic Examination

An ophthalmic examination of 20 Shetland Ponies (*N* = 9 mushroom, *N* = 11 non-mushroom) was performed, and all 20 ponies were genotyped for all known coat color variants (Table 6). The only anomalies identified after a complete ocular exam were hypopigmentation of the anterior and posterior uvea, persistent pupillary membranes (PPMs) of the iris, and cysts of the corpora nigra (Table S3). An association between tobiano and hypopigmentation of the posterior uvea was identified (Fisher exact test, *P* = 2.2 × 10^−3^). Additionally, the cream allele was associated with hypopigmented anterior uvea (Fisher exact test, *P* = 2.6 × 10^−2^). Therefore, to evaluate the role of mushroom while controlling for other pigmentation variables a Bayesian approach was utilized. The models investigating the effect of tobiano and cream support the finding from Fisher’s exact testing. Specifically, tobiano was a significant predictor for hypopigmentation of the posterior uvea and one copy of the cream allele was found to be a potential explanation for hypopigmentation of the anterior uvea (Figure S2). These findings warrant further investigation in a larger sample set of individuals. An association of mushroom with any of the ocular anomalies observed was not detected. Further, all models analyzed do not support homozygosity for mushroom as a sufficient explainer of hypopigmentation in either the anterior or posterior uvea (Figure S2).

## 4. Discussion

Our initial GWAS approach, utilizing a relatively small sample set of well phenotyped horses, identified a single locus on ECA7 reaching genome wide significance and a shared 3 Mb homozygous haplotype in all mushroom ponies. Analyzing whole genome sequencing data from this region identified one variant in the mushroom pony sequenced, absent in 87 other horses. This variant in *MFSD12* (c.600dupC) was perfectly concordant with phenotype in 96 Shetland Ponies and was absent from all other breeds tested (*N* = 251), with the exception of the Miniature Horse which is frequently crossed with Shetland Ponies. 

*MFSD12* has been reported to be highly expressed in melanocytes relative to other cell types including keratinocytes [32], is highly conserved across vertebrates and plays an integral role in trafficking transmembrane solutes [33]. Recent studies have identified *MFSD12* as an important factor in melanogenesis, specifically in determining the quantity of eumelanin and pheomelanin produced in various human populations [32,33]. Specifically, *MFSD12* has been shown to play a role in suppressing eumelanin production while being required to produce pheomelanin [33]. On the other hand, a reduction in *MFSD12* expression leads to an increase in eumelanin content in melanocytes [33]. Variants in both the coding region and regulatory regions of *MFSD12* have been implicated in affecting intensity of pigmentation in African human populations. Two variants within *MFSD12,* rs56203814 and rsl0424065, have only been found in African populations, specifically in East Africa, and are associated with darker skin pigmentation [33]. Two variants upstream of *MFSD12,* rsll2332856 and rs6510760, are similarly associated with darker pigmentation, while the ancestral alleles at these loci are nearly fixed in populations with lighter skin such as Europeans and East Asians [33]. It is hypothesized that these variants decrease gene expression of *MFSD12* resulting in increased production of eumelanin. Variants in *MFSD12* have also been shown to play a role in the skin pigmentation of Eurasian populations showing a correlation with solar radiation/UV exposure [32]. Specifically, the missense variant rs2240751 has been identified as being specific to East Asian and Native American populations and is associated with lighter skin pigmentation. This variant (p.Tyr182His) is hypothesized to impair protein function and thus pigmentation [32]. 

The role of *MFSD12* in coat color pigmentation has also been further evaluated utilizing *MFSD12* knockout mice which have a complete absence of pheomelanin in their hair shaft when compared to the wildtype agouti mouse [33]. Similarly, a strain of mouse known as the “grizzled” mouse, has a dilute grey coat color with a reduction of pheomelanin in the hair shaft caused by a 9 bp deletion in exon 2 of *MFSD12*, (p.Leu163_Ala165del) [33]. Most recently in dogs, a missense variant in *MFSD12* (p.Arg51Cys) was identified as the likely causal variant for diluting pheomelanin to a light, cream color [34]. 

Given the perfect concordance with phenotype in Shetland Ponies and the role of *MFSD12* mutants in pigmentation variation reported for human, mice, and dogs, we propose that *MFSD12* c.600dupC is the causal variant for mushroom. If translated, this frameshift variant truncates 277 amino acids, and is thus likely deleterious to protein function. Homozygous *Mu/Mu* ponies show a dilution in pheomelanin as evidenced by the dilute coat. Though the mushroom dilution was originally reported to affect only chestnut ponies, we identified six *Mu/Mu* bay ponies, that show an altered phenotype that we describe here as bay mushroom. These ponies lack the reddish hue of a typical bay coat with an intriguing countershading on the top line (darker head, neck, shoulders, Figure 3). One of these six ponies was also heterozygous at the cream locus and displayed a significantly more dilute phenotype than the other bay mushroom ponies, suggesting an additive effect of the cream and mushroom dilution on a bay background. Additionally, five chestnut *Mu/Mu* ponies were heterozygous for cream (palomino). These ponies were not observed to be significantly more dilute than *Mu/Mu* ponies without any copies of the cream dilution (Figure 4). However, interestingly, these ponies also had darker faces. We hypothesize that the truncated protein results in a decrease in pheomelanin production as evidenced by both dilute chestnut and bay horses. Similar to what has been shown in humans, we also hypothesize that that altered *MFSD12* increases eumelanin production resulting in the darker countershading on bay horses. Quantification of both eumelanin and pheomelanin content in mushroom and non-mushroom ponies of diverse coat color backgrounds is necessary to test these hypotheses further. This evaluation will also help to uncover the possible interactions between *SLC45A2* and *MFSD12* and their ultimate impact on phenotype.

As evidenced by several studies in the horse, ocular anomalies are a pleiotropic effect of pigmentation variants [9,35,36,37]. We therefore also aimed to determine if the mushroom allele was associated with any ocular anomalies. Examining nine mushroom ponies did not identify any consistent ocular anomalies present in mushroom ponies compared to non-mushroom ponies. While hypopigmentation of the posterior uvea and anterior uvea was noted in four and six of the mushroom ponies respectively, only the tobiano allele, and the cream allele were identified by our Bayesian models to explain this hypopigmentation. Ponies with the tobiano allele were more likely to have hypopigmentation of the posterior uvea and ponies with at least one copy of the cream allele were more likely to have hypopigmentation of the anterior uvea. Examining additional horses with these coat color alleles across breeds should help to substantiate this finding. 

A GWAS and subsequent whole genome sequencing approach identified a single variant perfectly concordant with the mushroom phenotype described in Shetland Ponies (*MFSD12* c.600dupC). Screening additional ponies also identified a new phenotype, specifically bay mushroom, that can be explained by homozygosity for the mushroom allele on a bay background. This variant can be used in marker-assisted selection in breeding programs selecting for or away from the mushroom coat color phenotypes.

## Figures and Tables

**Table 1:** Eight SNPs reaching genome wide significance and spanning the 326 kb mushroom associated haplotype.

Location ^1^	Variant ID	GWAS P-Value ^2^	*P*_combined_-Value (genotype) ^3^
chr7:3290682	rs68661375	2.08 × 10^−10^	9.84 × 10^−17^
chr7:3293765	rs395756529	2.08 × 10^−10^	9.84 × 10^−17^
chr7:3305324	rs395273754	2.08 × 10^−10^	2.71 × 10^−16^
chr7:3431918	rs1139100844	2.08 × 10^−10^	9.84 × 10^−17^
chr7:3459389	rs68664417	2.87 × 10^−9^	5.20 × 10^−15^
chr7:3468232	rs68664452	2.08 × 10^−10^	9.84 × 10^−17^
chr7:3482185	rs1148601322	2.08 × 10^−10^	9.84 × 10^−17^
chr7:3616751	rs394856954	1.16 × 10^−9^	9.84 × 10^−17^

^1^ SNPs based on Equcab 3 assembly. ^2^ Association from GWAS SLMM (*N* = 24). ^3^ Association under a recessive model of combined GWAS sample set and validation sample set (*N* = 69).

**Table 2:** ECA7 326 kb mushroom associated haplotype data across breeds. The mushroom associated haplotype was genotyped in the Shetland Pony and four additional breeds.

Breed/Phenotype	Homozygous Reference	Heterozygous	Homozygous Alternate
Shetland (Mu)^1^	0	0	40
Shetland (Ch)^2^	14	15	0
Shetland/^3^	30	3	0
Miniature Horse/^4^	24	4	1
Icelandic Horse/^4^	19	8	2
Quarter Horse/^4^	16	6	0
Thoroughbred/^4^	16	8	0

^1^ Mushroom Shetland Ponies. ^2^ Chestnut Shetland Ponies. ^3^ These samples were used to estimate the frequency of the mushroom associated haplotype in the Shetland Pony. 33 unrelated Shetland Ponies were investigated and coat color phenotype of this sample set was not considered in this analysis. ^4^ Unrelated horses from four additional breeds were used to determine if this haplotype was present in other breeds and to help identify horses that that could aid in prioritizing putative causal variants.

**Table 3:** Genotyping results for *MFSD12* c.600dupC in Shetland Ponies. Presented are results for the phenotyped sample set of Shetland Ponies (mushroom and chestnut) and the p-value for the mushroom associated variant.

	Genotype		
Phenotype	*N/N*	*N/Mu* ^1^	*Mu/Mu* ^1^
Shetland (Mu) ^2^	0	0	45
Shetland (Ch) ^3^	32	19	0
X^2^		*P* = 1.15 × 10^−22^	

^1^ Mu denotes the mushroom allele. ^2^ Mushroom Shetland Pony. ^3^ Chestnut Shetland Pony.

**Table 4:** Allele frequencies for *MFSD12* c.600dupC. Eight additional breeds as well as a random sample set of Shetland Ponies were screened for the causal mushroom variant and allele frequencies are reported here.

	Genotype				
Breed	*N/N*	*N/Mu*	*Mu/Mu*	Sample Size	Frequency ^3^
Shetland Pony ^1^	143	24	10	177	0.12
Miniature Horse	124	5	0	129	0.02
Icelandic Horse	29	0	0	29	0
Belgian	33	0	0	33	0
Rocky Mtn Horse ^2^	59	0	0	59	0
Friesian	32	0	0	32	0
Arabian	35	0	0	35	0
Quarter Horse	32	0	0	32	0
Thoroughbred	31	0	0	31	0

^1^ Random population of Shetland Ponies. ^2^ Rocky Mountain Horse. ^3^ Allele frequency for *MFSD12* c.600dupC.

**Table 5:** Bay, black and palomino ponies homozygous for *MFSD12* c.600dupC. Twelve Shetland Ponies were identified as being homozygous for the mushroom variant on a non-chestnut background.

Non-Chestnut Mushroom Ponies						
Individual	Agouti	Extension	Mushroom	Cream	Dun	Phenotype
Bay	*A/a*	*E/e*	*Mu/Mu*	*N/N*	*nd2/nd2*	dilute bay
Bay	*A/a*	*E/e*	*Mu/Mu*	*N/N*	*nd1/nd2*	dilute bay
Bay	*A/a*	*E/e*	*Mu/Mu*	*N/N*	*nd2/nd2*	dilute bay
Bay	*A/A*	*E/e*	*Mu/Mu*	*N/Cr*	*nd2/nd2*	buckskin dilute
Bay	*A/A*	*E/e*	*Mu/Mu*	*N/N*	*nd2/nd2*	dilute bay
Bay	*A/a*	*E/e*	*Mu/Mu*	*N/N*	*nd2/nd2*	dark dilute bay
Black	*a/a*	*E/e*	*Mu/Mu*	*N/N*	*nd2/nd2*	black
Palomino	*a/a*	*e/e*	*Mu/Mu*	*N/Cr*	*nd2/nd2*	mushroom
Palomino	*a/a*	*e/e*	*Mu/Mu*	*N/Cr*	*nd2/nd2*	mushroom
Palomino	*a/a*	*e/e*	*Mu/Mu*	*N/Cr*	*nd2/nd2*	mushroom
Palomino	*A/a*	*e/e*	*Mu/Mu*	*N/Cr*	*nd2/nd2*	mushroom
Palomino	*a/a*	*e/e*	*Mu/Mu*	*N/Cr*	*D/nd2*	dilute dunalino

**Table 6:** Ophthalmic Findings in Mushroom Ponies. A complete ocular exam was performed on twenty Shetland Ponies Genotypes for coat color with variation in the sample set are also reported.

Ophthalmic Findings								
Individual	*ASIP* ^1^	*MC1R* ^2^	*MFSD12* ^3^	*SLC45A2* ^4^	*KIT* ^5^	*MITF* ^6^	Anterior ^7^	Posterior ^8^
463	*a/a*	*e/e*	*Mu/Mu*	*N/Cr*	*N/To*	*N/S*	OD hypo ^11^	hypo ^9^
466	*a/a*	*e/e*	*Mu/Mu*	*N/N*	*N/To*	*N/N*	hypo	pig ^10^
467	*a/a*	*e/e*	*Mu/Mu*	*N/Cr*	*N/N*	*N/N*	hypo	pig
477	*a/a*	*e/e*	*Mu/Mu*	*N/N*	*N/N*	*N/N*	hyper	pig
478	*A/a*	*e/e*	*Mu/Mu*	*N/N*	*N/N*	*N/N*	hyper	pig
479	*a/a*	*e/e*	*Mu/Mu*	*N/N*	*N/N*	*N/N*	hypo	pig
480	*a/a*	*e/e*	*Mu/Mu*	*N/N*	*N/To*	*N/N*	pig	hypo
481	*a/a*	*e/e*	*Mu/Mu*	*N/N*	*N/To*	*N/N*	hypo	hypo
483	*a/a*	*e/e*	*Mu/Mu*	*N/N*	*N/To*	*N/N*	hypo	hypo
464	*A/a*	*e/e*	*N/Mu*	*N/Cr*	*To/To*	*N/S*	hypo	hypo
465	*A/A*	*e/e*	*N/Mu*	*N/N*	*N/To*	*N/N*	pig	pig
468	*a/a*	*e/e*	*N/Mu*	*N/N*	*N/To*	*N/N*	pig	hypo
469	*a/a*	*e/e*	*N/Mu*	*N/N*	*N/To*	*N/N*	pig	hypo
470	*a/a*	*E/e*	*N/Mu*	*N/N*	*N/To*	*N/N*	pig	hypo
471	*a/a*	*E/e*	*N/N*	*N/N*	*N/N*	*N/N*	pig	pig
472	*A/a*	*e/e*	*N/N*	*N/N*	*N/To*	*N/N*	pig	hypo
473	*A/a*	*E/e*	*N/N*	*N/N*	*N/To*	*N/N*	pig	hypo
474	*a/a*	*e/e*	*N/Mu*	*N/N*	*N/To*	*N/N*	hypo	pig
475	*A/a*	*e/e*	*N/Mu*	*N/Cr*	*N/To*	*N/N*	hypo	hypo
476	*A/A*	*E/e*	*N/N*	*N/N*	*N/N*	*N/N*	pig	pig

^1^ Agouti genotype. ^2^ MC1R (red factor) genotype. ^3^ Mushroom genotype. ^4^ Cream genotype. ^5^ Tobiano genotype. ^6^ Splash white 1 genotype. ^7^ Anterior uveal pigmentation. ^8^ Posterior uveal pigmentation. ^9^ hypopigmentation. ^10^ pigmented (normal). ^11^ This pony had one blue eye likely due to splash white 1.

## Supplementary Materials

The following are available online at https://www.mdpi.com/2073-4425/10/10/826/s1, Figure S1: Investigating Genomic Inflation in the GWAS Sample Set. Identity by descent and Population structure, Figure S2: Regression Coefficient Plots, Table S1: Primer sequences for eight top prioritized GWAS SNPs. Genotyping was performed by Agena Bioscience MassArray technology, Table S2: Primer sequences for Sanger sequencing and confirming *MFSD12* (c.600dupC), Table S3: Ophthalmic Findings and Coat Color Genotypes.
