# Supplementary material for: Frameshift Variant in MFSD12 Explains the Mushroom Coat Color Dilution in Shetland Ponies
Source: Genes (Basel). 2019 Oct 19;10(10):826. doi: 10.3390/genes10100826 (PMC6827053; doi:10.3390/genes10100826)
Supplement: Supplementary file 1 [file genes-10-00826-s001.pdf]

Frameshift Variant in *MFSD12* Explains the Mushroom Coat

Color Dilution in Shetland Ponies

Jocelyn Tanaka[1], Tosso Leeb[2], James Rushton[3], Thomas R. Famula[4], Maura Mack[1], Vidhya Jagannathan[2], Christine Flury[5], Iris Bachmann[6], John Eberth[7], Sue M. McDonnell[8], Maria Cecilia T. Penedo[1], and Rebecca R. Bellone[1,9]

[1] Veterinary Genetics Laboratory, School of Veterinary Medicine, University of California, Davis, CA 95616

[2] Institute of Genetics, University of Bern, 3001 Bern, Switzerland

[3] Rowe Equine Ltd, Wotton Under Edge, GLOS, GL12 7PP

[4] Department of Animal Science, University of California, One Shields Ave, Davis, CA 95616, USA

[5] School of Agricultural Forest and Food Sciences, Bern University of Applied Sciences, 3052 Zollikofen, Switzerland

[6] Agroscope, Swiss National Stud Farm, 1580 Avenches, Switzerland

[7] University of Kentucky College of Agriculture, Food and Environment , Lexington, KY 40546

[8] University of Pennsylvania School of Veterinary Medicine, Philadelphia, PA 19104

[9] Population Health and Reproduction, School of Veterinary Medicine, University of California,

Davis, CA 95616, USA

Supplemental Tables and Figures

Table S1 Primer sequences for eight top prioritized GWAS SNPs. Genotyping was performed using Agena Bioscience MassArray technology.

MassArray Marker Assayed	Variant ID	Association*	Primer	Primer Sequence
7:718169	rs396258064	**2.15 X 10-5**	**2nd-PCRP****	ACGTTGGATGCATCTGCCCTCGTTTCTATC
			**1st-PCRP****	ACGTTGGATGCGAGGTGCAAAATGACAAGG
			**UEP_SEQ*****	GCTGTTTCTGAGTTGGC
7:3290682	rs68661375	**9.84 X 10-17**	**2nd-PCRP**	ACGTTGGATGATGAGCCAGGTGGGTGTTTC
			**1st-PCRP**	ACGTTGGATGCTGCCTGACGTTCTAGAATG
			**UEP_SEQ**	GCGCCGGCCCCGCAC
7:3293765	rs395756529	**9.84 X 10-17**	**2nd-PCRP**	ACGTTGGATGGACCTTGACTGAAACGCTTG
			**1st-PCRP**	ACGTTGGATGAAAGTCAAAGAGCTCCACCC
			**UEP_SEQ**	AACGCTTGAGGCGAG
7:3305324	rs395273754	**2.71 X 10-16**	**2nd-PCRP**	ACGTTGGATGAAGAAAGCCGACTGTGGAAG
			**1st-PCRP**	ACGTTGGATGCTGTAAATGCCTCGCCGAAG
			**UEP_SEQ**	ACTGTGGAAGACATTAATTACTC
7:3431918	rs1139100844	**9.84 X 10-17**	**2nd-PCRP**	ACGTTGGATGGCAATGCTCGGTCCCAAAC
			**1st-PCRP**	ACGTTGGATGTCCAAACGGCGTTTCCAAAG
			**UEP_SEQ**	CGCATCGTCTCCAAATT
7:3459389	rs68664417	**5.20 X 10-15**	**2nd-PCRP**	ACGTTGGATGACGCTTTCTGTCGAACCATC
			**1st-PCRP**	ACGTTGGATGTTTGTCGGTGTCTTTGAGGG
			**UEP_SEQ**	CGAACCATCTTAGCCC
7:3468232	rs68664452	**9.84 X 10-17**	**2nd-PCRP**	ACGTTGGATGCGACCCCACACGAAGCCAC
			**1st-PCRP**	ACGTTGGATGTGGTTCCATATGGTGATACG
			**UEP_SEQ**	CCCCGAAGCCACCCCTGG
7:3482185	rs1148601322	**9.84 X 10-17**	**2nd-PCRP**	ACGTTGGATGGGTCCCTAAAGACTCATTGG
			**1st-PCRP**	ACGTTGGATGTGGAGCACTTTGCCCATCAC
			**UEP_SEQ**	CCCCGGTTGCAGGAAACTCAC
7:3616751	rs394856954	**9.84 X 10-17**	**2nd-PCRP**	ACGTTGGATGGCAAGGAACAGCAATTCAGC
			**1st-PCRP**	ACGTTGGATGAAAGGGACAGTCAGAGTGAG
			**UEP_SEQ**	TGTGTGTAAAAACGTGGACC
7:3749780	rs68666954	**1.53 X 10-5**	**2nd-PCRP**	ACGTTGGATGTTGCATACTGTGCCCAGATG
			**1st-PCRP**	ACGTTGGATGTTCCAGTTCATCCGCCGCTC
			**UEP_SEQ**	CCCAGATGCATCTTGGGAA

* Association calculated under a recessive model. ** 2nd and 1st PCRP denotes the forward and reverse primers. *** UEP_Seq denotes extension primer.

Table S2 Primer sequences for Sanger sequencing *PLIN3* and confirming *MFSD12* (c.600dupC)

Primer Name	Forward primer	Reverse primer
MFSD12 Variant	GCTCACCCGGAACACCGGCA	ACCGCCAGTCTTGCGCCATC

Table S3 Ophthalmic Findings and Coat Color Genotypes for those Shetland Ponies Examined

Ophthalmic Findings										
Individual	ASIP[1]	MC1R[2]	Mu[3]	Cr[4]	To[5]	SW1[6]	Anterior[7]	Posterior[8]	Iris PPM[11]	Corpora Nigra Cyst
463	*a/a*	*e/e*	*M/M*	*N/C*	*N/T*	*N/S*	OD hypo	hypo*		OD[10] cysts
466	*a/a*	*e/e*	*M/M*	*N/N*	*N/T*	*N/N*	hypo	pig**		
467	*a/a*	*e/e*	*M/M*	*N/C*	*N/N*	*N/N*	hypo	pig	OU[9]: iris-iris PPMs	
477	*a/a*	*e/e*	*M/M*	*N/N*	*N/N*	*N/N*	hyper	pig		
478	*A/a*	*e/e*	*M/M*	*N/N*	*N/N*	*N/N*	hyper	pig	OU: iris-iris PPMs	
479	*a/a*	*e/e*	*M/M*	*N/N*	*N/N*	*N/N*	hypo	pig	OU: iris-iris PPMs	
480	*a/a*	*e/e*	*M/M*	*N/N*	*N/T*	*N/N*	pig	hypo		
481	*a/a*	*e/e*	*M/M*	*N/N*	*N/T*	*N/N*	hypo	hypo	OU: iris-iris PPMs	
483	*a/a*	*e/e*	*M/M*	*N/N*	*N/T*	*N/N*	hypo	hypo		
464	*A/a*	*e/e*	*N/M*	*N/C*	*T/T*	*N/S*	hypo	hypo		
465	*A/A*	*e/e*	*N/M*	*N/N*	*N/T*	*N/N*	pig	pig		
468	*a/a*	*e/e*	*N/M*	*N/N*	*N/T*	*N/N*	pig	hypo		
469	*a/a*	*e/e*	*N/M*	*N/N*	*N/T*	*N/N*	pig	hypo		
470	*a/a*	*E/e*	*N/M*	*N/N*	*N/T*	*N/N*	pig	hypo		
471	*a/a*	*E/e*	*N/N*	*N/N*	*N/N*	*N/N*	pig	pig	OU: iris-iris PPMs	OU cysts
472	*A/a*	*e/e*	*N/N*	*N/N*	*N/T*	*N/N*	pig	hypo		
473	*A/a*	*E/e*	*N/N*	*N/N*	*N/T*	*N/N*	pig	hypo	OU: iris-iris PPMs	
474	*a/a*	*e/e*	*N/M*	*N/N*	*N/T*	*N/N*	hypo	pig		
475	*A/a*	*e/e*	*N/M*	*N/C*	*N/T*	*N/N*	hypo	hypo	OU: iris-iris PPMs	
476	*A/A*	*E/e*	*N/N*	*N/N*	*N/N*	*N/N*	pig	pig	OU: iris-iris PPMs	

[1] Agouti genotype. [2] MC1R (red factor) genotype. [3] Mushroom genotype. [4] Cream genotype. [5] Tobiano genotype. [6] Splash white 1 genotype. [7] Anterior uveal pigmentation. [8] Posterior uveal pigmentation. [9] OU refers to both eyes. [10]OD refers to the right eye. [11]PPMs are persistent pupillary membranes. *hypopigmentation. **pigmented (normal). *** This pony had one blue eye likely due to splash white 1.

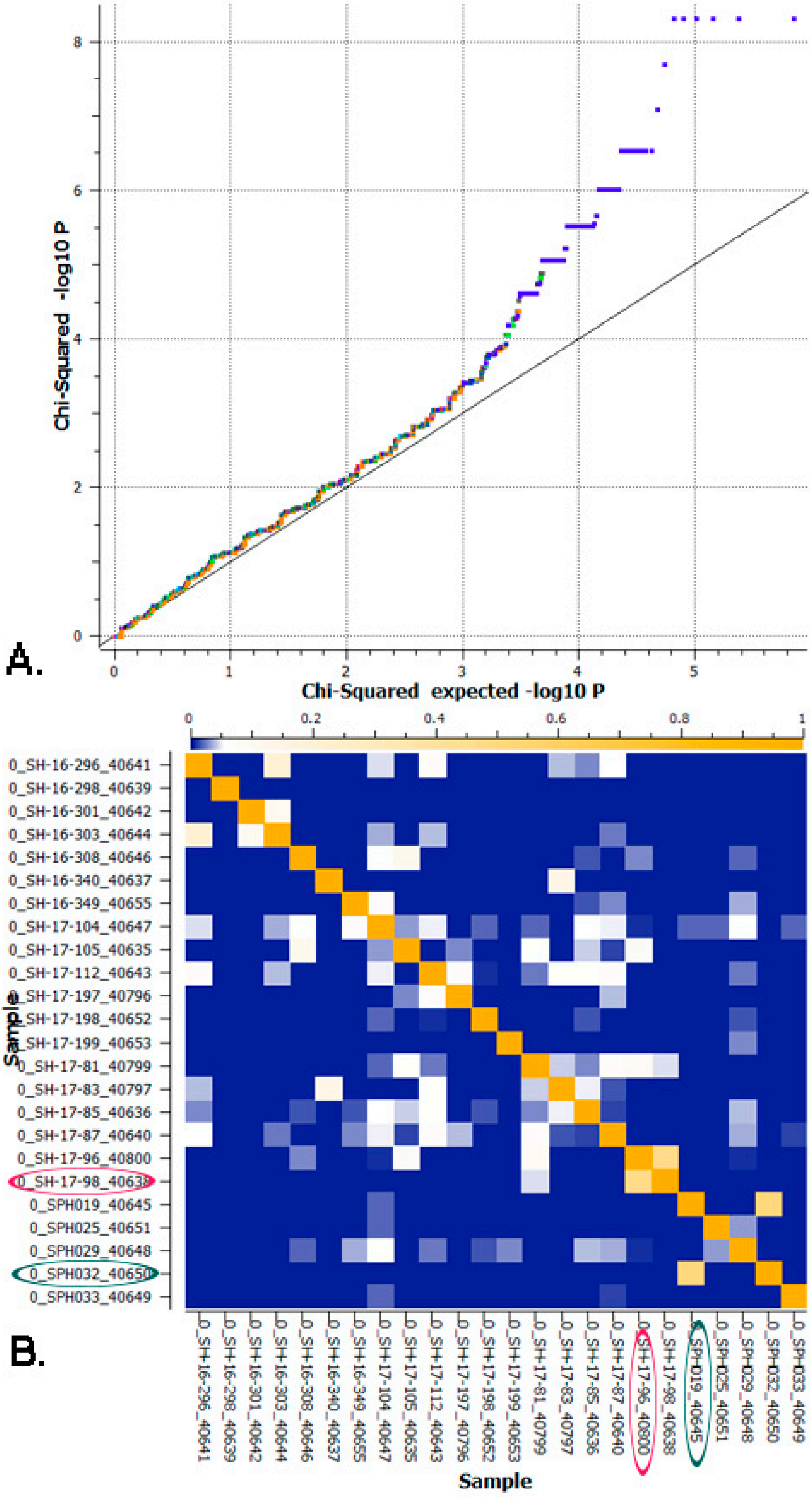
A.
Chi-Squared -log10 P
0
2
4
6
8
Chi-Squared expected -log10 P
0
1
2
3
4
5
B.
0
0.2
0.4
0.6
0.8
1
Sample
0_SH-16-296_40641
0_SH-16-298_40639
0_SH-16-301_40642
0_SH-16-303_40644
0_SH-16-308_40646
0_SH-16-340_40637
0_SH-16-349_40655
0_SH-17-104_40647
0_SH-17-105_40635
0_SH-17-112_40643
0_SH-17-197_40796
0_SH-17-198_40652
0_SH-17-199_40653
0_SH-17-81_40799
0_SH-17-83_40797
0_SH-17-85_40636
0_SH-17-87_40640
0_SH-17-96_40800
0_SH-17-98_40638
0_SPH019_40645
0_SPH025_40651
0_SPH029_40648
0_SPH032_40650
0_SPH033_40649
Sample

Figure S1: Investigating Genomic Inflation in the GWAS Sample Set. Identity by descent and Population structure

A) Q-Q plot of chi-squared -log10 P vs chi-squared expected -lop10 P calculated from a χ^2 basic allelic association test. Markers on ECA7 are represented in indigo. B) Heat map displaying identity by descent (IBD) in the sample set. Two pairs of samples (circled in pink and teal) shared up to 52% of their genome. Intensity of yellow shading on the heat map denotes level of genome showing.

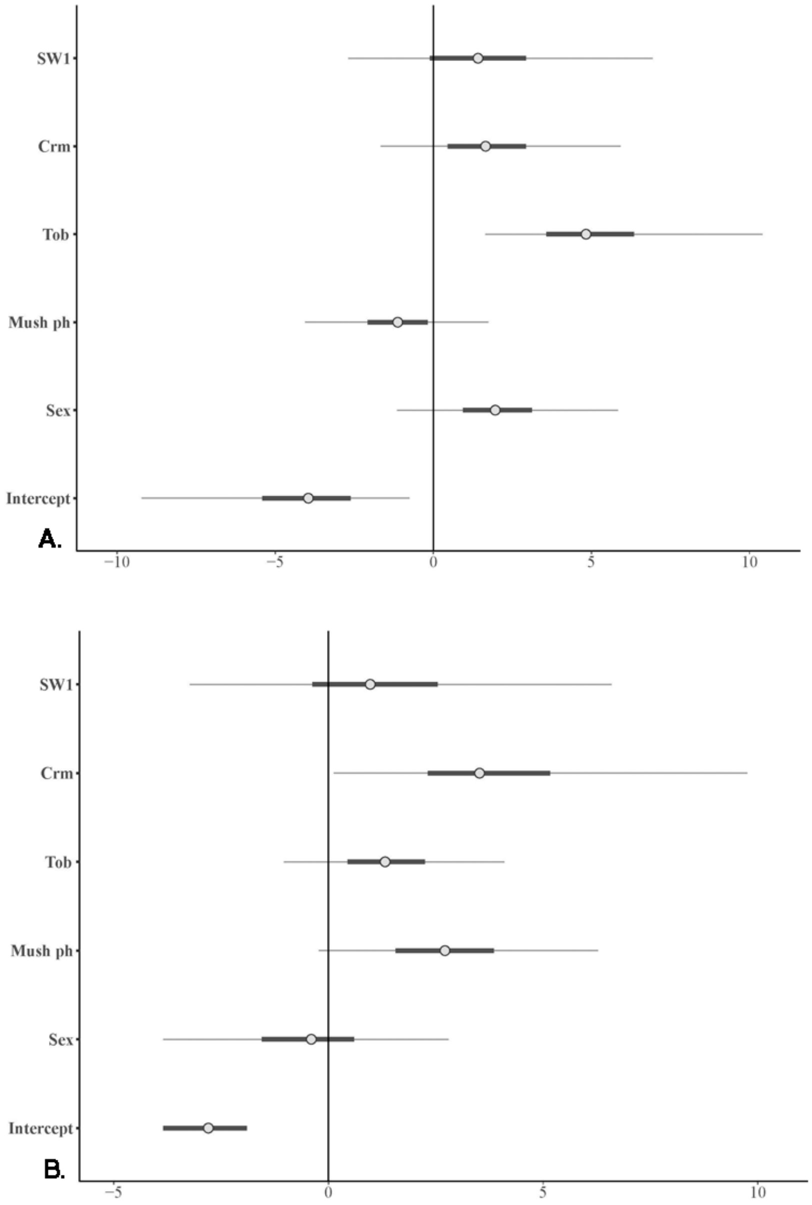

Figure S2: Regression Coefficient Plots for Potential Variables Explaining Posterior and Anterior Uveal Pigmentation Variation.

Both plots show the regression coefficients considering sex, mushroom phenotype (mush_ph), tobiano phenotype (tob), cream phenotype (crm), and splash white 1 phenotype (SW1) for posterior (A) and anterior (B) uveal pigmentation. The circles are an estimate of the coefficient, the thick line represents a 50% confidence interval and the thin line represents a 95% confidence interval. A) Regression coefficient plot for posterior uveal pigmentation. Tobiano looks to be the only variable tested that could explain hypopigmentation of the posterior uvea. B) Regression coefficient plot for anterior uveal pigmentation. The cream allele is the only variable tested explaining hypopigmentation of the anterior uvea.
